# Relationship of spinal alignment with muscular volume and fat infiltration of lumbar trunk muscles

**DOI:** 10.1371/journal.pone.0200198

**Published:** 2018-07-05

**Authors:** Rafael Menezes-Reis, Gustavo Perazzoli Bonugli, Carlos Ernesto Garrido Salmon, Debora Mazoroski, Carlos Fernando Pereira da Silva Herrero, Marcello Henrique Nogueira-Barbosa

**Affiliations:** 1 Ribeirão Preto Medical School—University of São Paulo, Ribeirão Preto, Brazil; 2 Ribeirão Preto Philosophy and Sciences School—University of São Paulo, Ribeirão Preto, Brazil; Medical College of Wisconsin, UNITED STATES

## Abstract

Fat infiltration and atrophy of lumbar muscles are related to spinal degenerative conditions and may cause functional deficits. Spinal alignment exerts biomechanical influence on lumbar intervertebral discs and joints. Our objective was to evaluate if spinopelvic parameters correlate with the lumbar muscle volume and fat infiltration. This is an observational, prospective and cross-sectional study. Ninety-three asymptomatic adult aged 20–40 years were included. Lumbar lordosis (LL), thoracic kyphosis (TK), pelvic incidence (PI), pelvic tilt (PT), sacral slope (SS), thoracolumbar alignment (TL), sagittal vertical axis (SVA), C2-pelvic angle (CPA), spinosacral angle (SSA), lack of lordosis (PI-LL), L1S1 and T1S1 length were measured on panoramic spine radiographs. Lumbar axial T1-weighted and In- and Out-Phase images were obtained on 1.5T MRI scanner and were used to extract the muscle volumes and fat fractions of multifidus, erector spinae, and psoas. All muscle volumes were higher in men than women (p<0.05). The fat fraction was higher in the multifidus and erector spinae in women (p<0.05). Multifidus volume was weakly correlated with PT (R = 0.22), PI (R = 0.22), LL (R = 0.34) and CPA (R = 0.29). Erectors spinae volume were correlated with CPA (R = 0.21). Psoas volume correlated with TK (R = 0.21), TL (R = 0.27) and SVA (R = -0.23). The lumbar muscle volumes showed a moderated correlation with T1S1 length (R = 0.55 to 0.62). Spinopelvic parameters showed correlation with lumbar muscle volumes but not with muscle fat infiltration on asymptomatic young adults.

## Introduction

Fat infiltration of skeletal muscles are related to spinal degenerative conditions, may cause functional deficits in skeletal muscles and negatively affect the contractility because muscle fibers are replaced by non-contractile tissue [1]. Recently, the study of spine muscles composition has been the target of several authors. Aging has been presented as a powerful influencing factor in increasing fatty infiltrate even in asymptomatic subjects [2]. Trophic changes and intramuscular fat infiltration have been observed on various lumbar spine diseases like disc herniation [3], lumbar spinal stenosis [4], unilateral radiculopathy [5], and sway back posture [6]. Although, lumbar lordosis reduction is associated with a significant loss of paraspinal muscle mass and a consequent increase of fat infiltration [7]. Furthermore, deteriorated muscle composition may contribute to the recurrence of low back pain (LBP) [8] and there is few evidence about any association with future LBP and physical performance outcomes [9].

The balanced spinal alignment optimizes muscle energy expenditure and joint stress, where the gravity center is in a physiological position and able to maintain static and dynamic postures [10]. According to the concept of spinal balance on the sagittal plane, the spine must be aligned with the pelvis and the sacrum for the standing posture to be mechanically efficient [11]. Pelvic retroversion is developed during the neuromotor developmental transition from crawling to standing and is greatly influenced by the lumbar erector muscles. During this process, the sacrum tends to become more vertical due to the action of the lumbosacral muscles, multifidus and lumbar iliocostalis, and there is an increase in the sacral slope (SS) and pelvic tilt (PT) [10]. Optimal spine alignment depends on a synergistic relationship between the pelvic morphology and spinal curvature [11,12].

The forces applied by lumbar extensor muscles should be greater in spines with larger lumbar lordosis according to previous mathematical modeling [13]. Later Meakin et al tested this model using lumbar spine MRI, was they measured both extensor muscles and lumbar spine curvature based on the angle between the superior endplates of L4 and S1 vertebrae [14]. Their results support the hypothesis that lumbar extensor muscle volume is related to the magnitude of the sagittal curvature. However, that study has significant limitations, since lumbar lordosis was not measured in the upright physiologic position, the study included only female volunteers, and other spinopelvic parameters were not evaluated. Additionally, paraspinal muscle volume affects the stability of the spinal segments [15].

Several spinopelvic parameters have been described and used in the recent literature, being used to classify and categorize the spinal alignment in the sagittal plane [11,12]. Spinopelvic parameters may be associated with degenerative disc disease, hip and facet joint osteoarthritis [16,17].

To our knowledge, there is lacking information regarding the correlations between spinal alignment and paraspinal musculature morphometry and fat infiltration in healthy subjects. Our hypothesis is that different postural spine alignments and measurable spinopelvic parameters could be correlated with extensor muscle morphological changes.

## Materials and methods

This study was an observational, prospective cross-sectional study approved by the Institutional Review Board of the University Hospital, Ribeirao Preto Medical School, USP (protocol number 4187/2013). The volunteers were verbally informed about the objectives and were included only after reading and signing the written informed consent statement.

### Volunteers

We recruited 93 asymptomatic adult volunteers aged 20–40 years (43 men and 50 women). The inclusion criteria for the study were an absence of LBP during the preceding six months, an Oswestry Dysfunction Index score <10 and sedentary or low levels of physical activity as characterized by the International Physical Activity Questionnaire. The exclusion criteria were: volunteers with persistent low back pain for more than three months, history of radiculopathy, neuromotor disorders, facet or hip osteoarthritis, previous spine and hip surgery, significant scoliosis, spine or hip fracture, osteoporosis and pregnant.

The mean age of the volunteers was 27.09±5.3 years (20–40 years). The anthropometric measurements of the subjects were as follows: height: 1.70±0.09 m (1.46–1.89 m), weight: 66.8±13.4 kg (44–105 kg), and body mass index (BMI): 23.0±3.3 kg/m^2^ (15.9–32 kg/m^2^).

### Spinopelvic parameter evaluation

Panoramic spine radiographs were obtained in the lateral view (Kodak Direct View, Carestream Health, Rochester, NY, USA), and the field of view (FOV) spanned from the internal acoustic meatus to the femoral heads, thereby allowing the entire spine to be studied. The assessment was performed using a 2m focus-film distance; the volunteers were in a standing position, their shoulders were flexed at 30°, and their elbows were flexed at approximately 90° [18].

**Fig 1:**
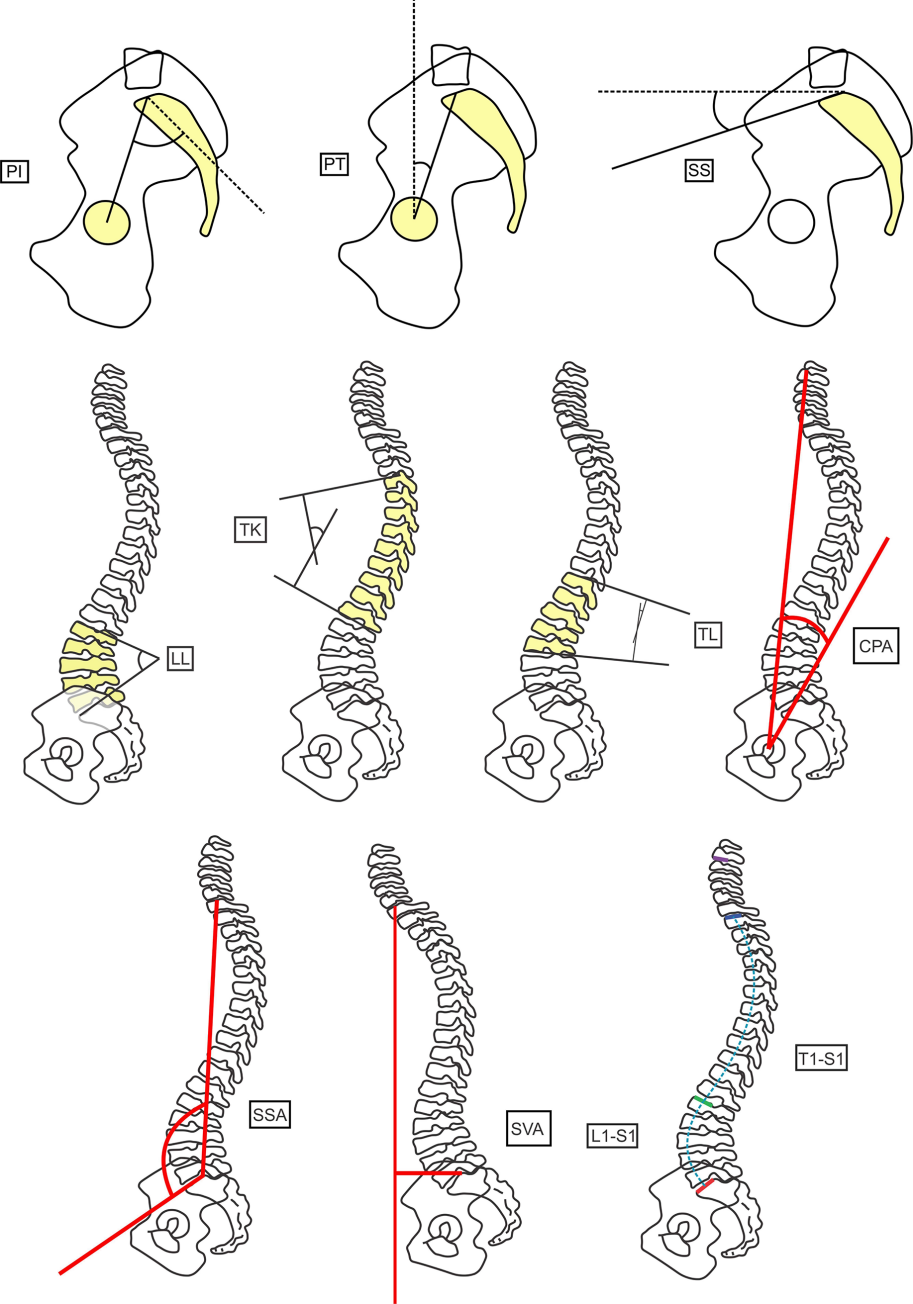
Spinopelvic parameters measured. Superior line: pelvic incidence—PI (*left*); pelvic tilt—PT (*center*); sacral slope—SS (*right*). Middle line: lumbar lordosis—LL (*left*); thoracic kyphosis—TK (*left-middle*); thoracolumbar alignment—TL (*middle-right*); C2 pelvic angle—CPA (*right*). Inferior line: spinosacral angle—SSA (*left*); sagittal vertical axis—SVA (*center*); L1S1 and T1S1 length (*right*).

**Fig 2:**
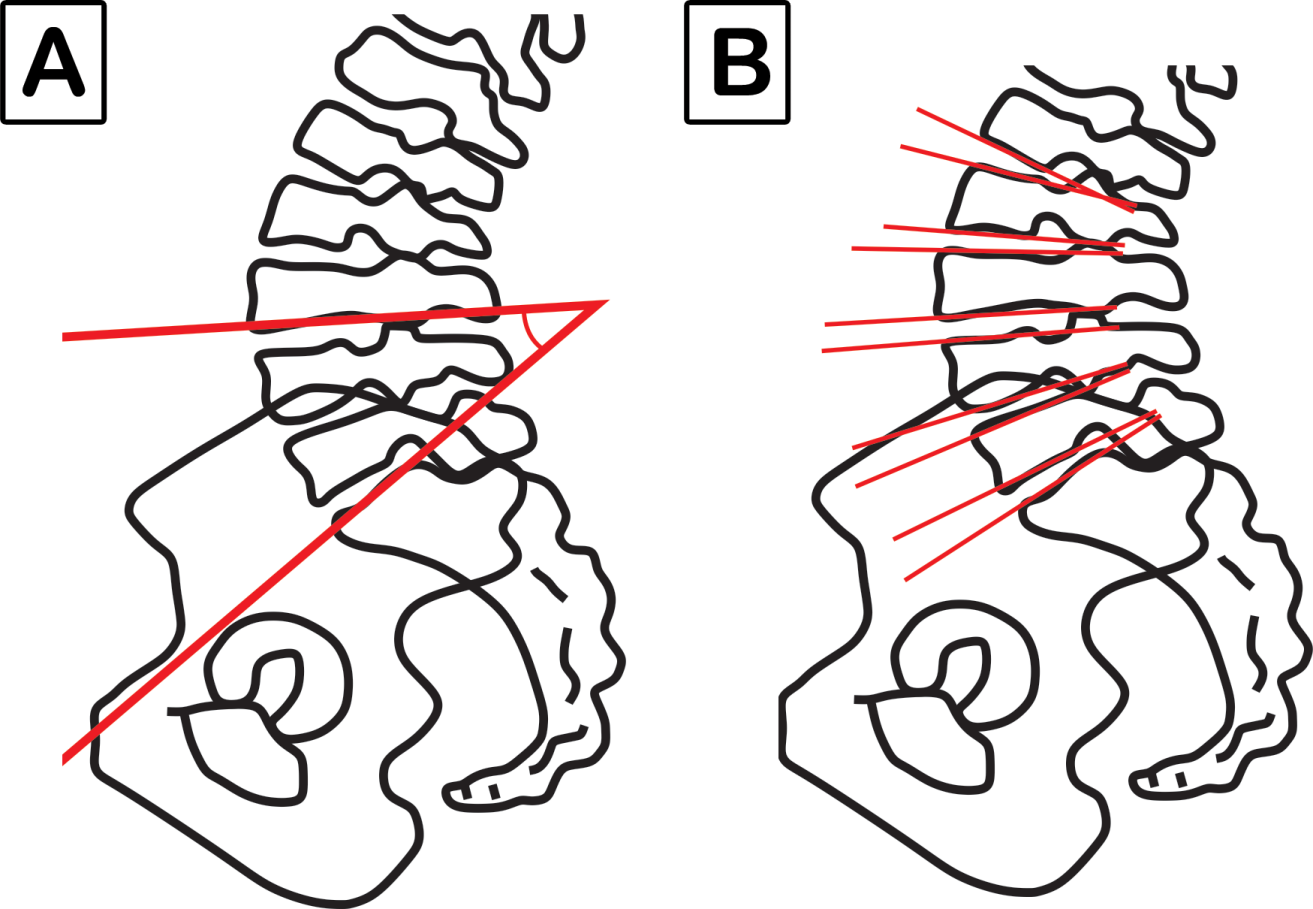
A) Lumbar lordosis angle measured by L3 inferior endplate to sacral endplate (L3-S1). B) Lumbar intervertebral angles: L3-L4, L4-L5, and L5-S1.

To measure the spinopelvic parameters in the panoramic radiographs, we used Surgimap® software (Nemaris Inc., New York, NY, USA). We analyzed the following parameters (Fig 1): SS, PT, pelvic incidence (PI), thoracic kyphosis (TK), thoracolumbar alignment (TL), sagittal vertical axis (SVA), spinosacral angle (SSA), C2 pelvic angle (CPA), lack of lordosis: defined as the subtraction between PI and LL (PI-LL), and the T1S1 and L1S1 lengths. For lumbar lordosis (LL), we use two analysis forms: 1) LL, the angle between the L1 upper vertebral endplate and sacral endplate; 2) LL: L3-S1, the angle between the L3 upper vertebral endplate and sacral endplate (Fig 2A). In addition, the intervertebral angles of L3-L4, L4-L5, and L5-S1 were also measured according to the methodology proposed by Viggiani et al [19] (Fig 2B).

### Muscle fat infiltration and volumetric evaluation

Lumbar spine MRI was performed using a 1.5T scanner (Achieva; Philips Healthcare, Best, Netherlands) with a 16-channel spinal coil. All MRI examinations were performed in the afternoon. Volunteers rested in a seated position for one hour prior to the scan. These precautions were followed to avoid daily physiological variations. The volunteers were positioned in a supine position, and their legs extended during the MRI exam.

T1-weighted images in the axial plane were acquired using the following parameters: spin echo sequence, TR 600 ms, TE 10 ms, flip angle 90°, FOV 180 x 180 mm, matrix 244 x 144, acquired voxel dimensions 1.0 x 1.0 x 4.0 mm, bandwidth 264 Hz/Px and slice thickness 4 mm. To determine the muscle fat fraction, we acquired in- and out-phase images using a gradient echo sequence with the same geometrical parameters as those used in the previous sequence to facilitate the image co-registration. The remaining acquisition parameters for this sequence were as follows: TR 232 ms, TE 2.3/4.6 ms, and a flip angle of 60°. The entire MRI protocol lasted 4 min and 18s.

**Fig 3:**
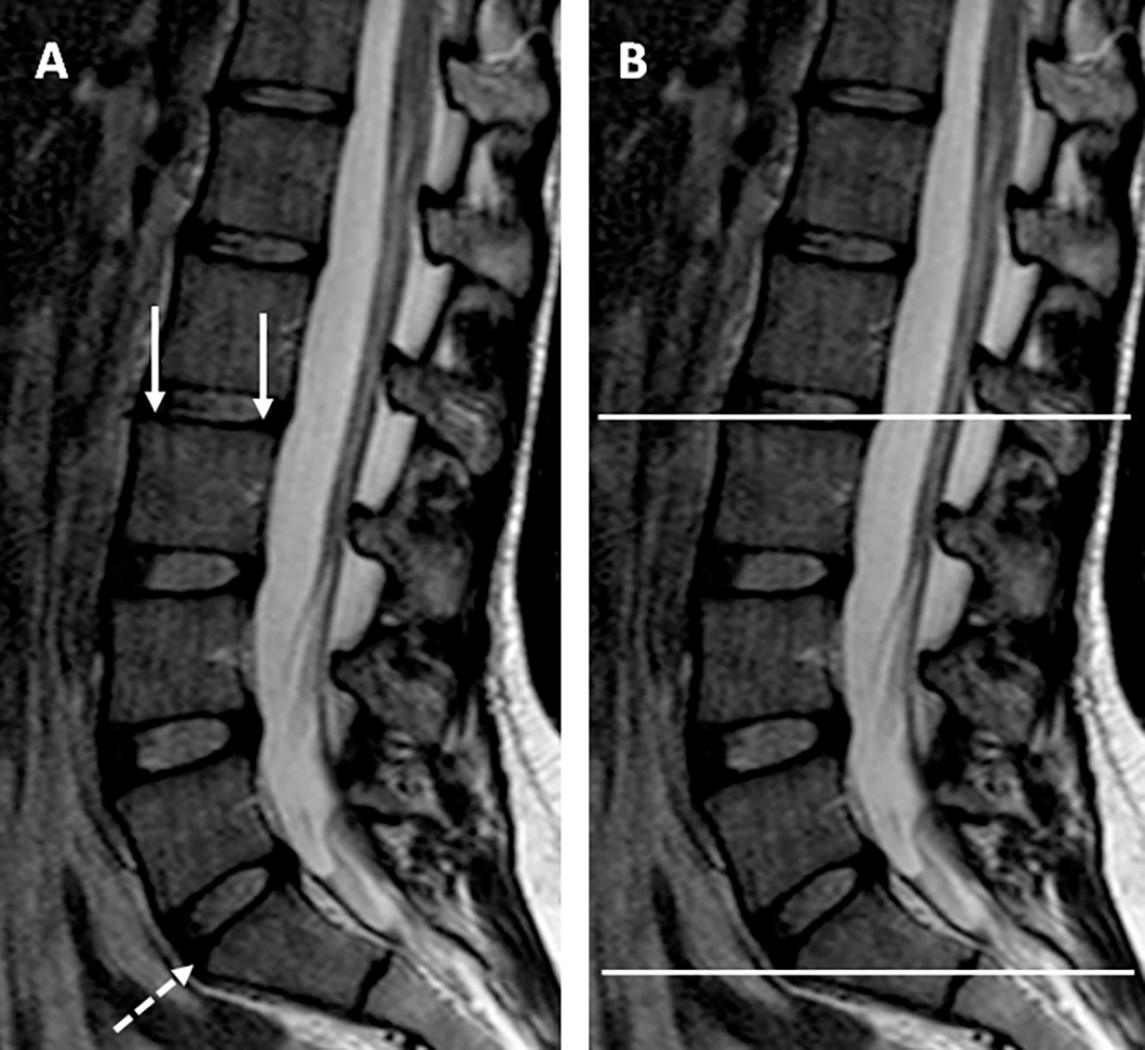
Sagittal T2-weighted image to demonstrate the superior and inferior limits of MRI axial slices acquisition. A) The white arrows point to the L3 superior endplate; the dashed arrow points the anterior margin of the S1 superior endplate. B) The white lines represent the upper and inferior limits of the block of axial slices.

**Fig 4:**
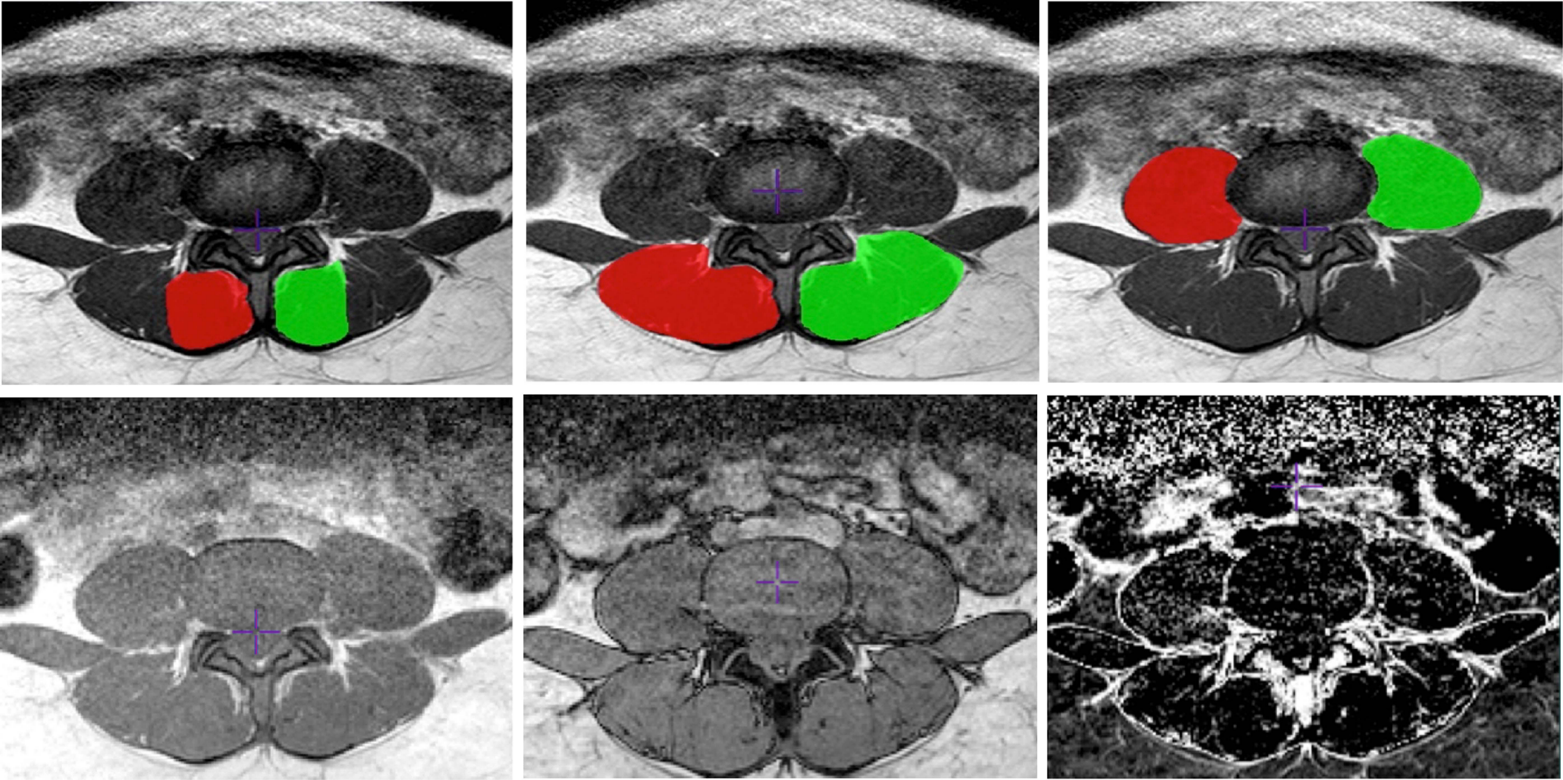
Superior line: Axial T1-weighted MRI at L4-L5 level with segmented lumbar muscles on Display® software. Right (green) and left (red) sides were indicated. *Left*: multifidus; *center*: erector spinae; *right*: psoas. Inferior line: Axial MRI sequences used to fat infiltration extraction. *Left*: In-Phase image; *center*: Out-Phase image; *right*: fat map.

Display® software and MINC tools (McConnell Brain Imaging Centre, Montreal, Quebec, Canada) were used for the manual segmentation and imaging analysis, respectively. The analysis was performed based on multiple axial T1-weighted images slices. To delimit the axial slices block, we used the L3 superior endplate as the upper limit; and the anterior margin of S1 superior endplate as the inferior limit (Fig 3). The contours of the multifidus, erector spinae, and psoas were manually traced in each slice to obtain the individual cross-sectional area (CSA) both for the right and left sides (Fig 4: superior line). At the end of the segmentation process, Display® software automatically generates a volumetric mask or label for each segmented muscle in a digital file. The muscle volume was obtained from this digital file.

The fat fraction (FF) was obtained using imaging techniques based on the difference in the chemical shift between water and fat due to the different precession frequencies of protons present in the water and fat molecules [20]. In our study, a fat peak at 1.3 ppm, which is related to bulk methylene protons, was selected as the main peak to represent the fat content. A pixel-by-pixel fat content map was generated from the two images (Fig 4: inferior line). This fat map is relative to the total proton content according to the expression below:

$$Fat\;Fraction\;Index\;(\%)=\frac{SI\;(ip)-\;SI(op)*100}{SI(ip)*2}$$

Where SI (ip) is the signal intensity of the in-phase image, and SI (op) is the signal intensity of the image in out-phase. The volumetric masks were co-registered and superimposed onto the fat maps to obtain the FF value in the region of interest in each analyzed muscle.

A researcher with three years of experience in spinal imaging analysis was responsible for the manual radiography and MRI measurements. A second examiner measured the same parameters independently to assess inter-observer agreement. The readers were blind to the clinical and MRI data.

### Statistical analysis

The Shapiro-Wilk test was used to test the normality of the data. Pearson's correlation (for parametric samples) or Spearman’s correlation (for nonparametric samples) were used to quantify the relationship among the spinopelvic parameters, height, weight and BMI and the data from the muscle volume and the fraction. For correlation coefficient (R) interpretation we adopt: zero to 0.20 as negligible, 0.20–0.40 as weak (or low), 0.40–0.60 as moderate, 0.60–0.80 as strong (or marked), 0.80–1.00 as high correlation [21]. GraphPad Prism v.5 (GraphPad Software, San Diego, CA, USA) and SPSS v.20 (IBM, Armonk, NY, USA) were used for the statistical analyses.

A significance level of 95% and statistical power of 80% test (β = 20%) were considered. The sample size was calculated using the correlation bivariate model, and 84 subjects were required, but we opted to increase the sample size to enhance the statistical power. We used G-Power v.3.1.7 (Universitat Kiel, Kiel, Germany) to calculate the sample size.

## Results

Table 1 presents the lumbar muscle volume and fat fraction values of the lumbar multifidus, erector spinae, and psoas. There was no statistical difference between the right and left multifidus volumes, but the left side FF average was 0.4 higher than in the right side (p = 0.0004). For lumbar erectors, the right side was 3.2 mm^3^ larger (p = 0.0012) and lumbar erectors FF was 0.4 higher on the right side (p<0.0001). The right psoas muscle volume was on average 1.52 mm^3^ higher than in the left side (p = 0.01), the fat fraction was similar on both sides (p = 0.30).

**Table 1:** Lumbar musculature morphometric measurements expressed as mean, standard deviation (SD) and range (minimum and maximum).

	Lumbar Multifidus		Lumbar Erectors		Psoas	
	*Mean/SD*	*Range*	*Mean/SD*	*Range*	*Mean/SD*	*Range*
Volume Right (mm^3^)	73.2±17.4	33.8–138.9	165.3±43.3 ^#^	92.3–282.8	102.7±35.7 ^#^	33.2–215.8
Volume Left (mm^3^)	72.7±16.7	38.8–125.1	162.1±44.2	92.5–287.1	101.2±36.3	27.5–208.1
Fat Fraction Right (%)	9.7±3.9	0.4–23.6	11.6±5.5 ^#^	0.4–30.1	7.2±3.8	0.14–24.47
Fat Fraction Left (%)	10.1±3.7 ^$^	0.8–22.5	12.0±5.5	0.11–30.3	7.3±3.8	0.73–23.9

$: the left side was significantly higher than the right side.

#: the right side was significantly higher than the left side.

Table 2 shows the CSA and fat fraction values of the muscles at three different intervertebral levels (L3-L4, L4-L5, and L5-S1). The lumbar multifidus at L5-S1 level had a higher fat fraction percentage than L4-L5 and L3-L4 levels (p = 0.001), and there was no significant difference between L4-L5 and L3-L4. Lumbar erectors fat fraction had a significant craniocaudal increase (L5-S1>L4-L5>L3-L4) (p < 0.001). Already for the psoas, the muscle section at L4L5 presented a significantly higher fat fraction than L5-S1 or L-3L4 levels (p < 0.001), but L5S1 and L3L4 were not statistically different. Regarding muscular cross-sectional areas, there were significant variations between each intervertebral level for multifidus (L5-S1>L4-L5>L3-L4), lumbar erectors (L3-L4>L4-L5>L5-S1) and psoas (L4-L5>L-3L4 and L5-S1). Our findings are in agreement with the expected muscle anatomy.

**Table 2:** Lumbar musculature morphometric measurements of cross sectional areas (mm^2^) and fat fraction (%) according to intervertebral level expressed as mean and standard deviation (SD).

	Lumbar Multifidus				Lumbar Erectors				Psoas			
	*CSA R*	*CSA L*	*FF R*	*FF L*	*CSA*. *R*	*CSA L*	*FF R*	*FF L*	*CSA R*	*CSA L*	*FF R*	*FF L*
L3-L4	12.91±4.2	12.3±3.5	8.6±7.3 #	9.2±7.1 #	47.1±4.5	42.9±11.5	7.2±2.9 #$	7.8±2.9 #$	18.9±7.1	18.6±7.1	6.2±5.4 $	6.4±5.2 $
L4-L5	17.6±3.7	17.4±4.0	8.4±3.1 #	8.8±3.2 #	39.6±8.5	40.1±8.1	9.2±3.1 #	9.8±3.2 #	23.1±7.7	22.9±7.6	8.4±3.1	8.9±3.2
L5-S1	18.1±8.5	17.1±5.3	9.9±3.1	9.8±2.9	23.7±7.7	23.4±8.2	11.3±3.4	11.0±3.2	18.7±7.9	19.0±7.7	6.0±1.9 $	6.2±1.8 $
Diff. between levels (p)	< 0.001		0.001		< 0.001		< 0.001		0.001		< 0.001	

CSA R:Cross-sectional area muscle right side, CSA L: Cross-sectional area muscle left side, FF R: fat fraction right side, FF L: fat fraction left side.

#: there was statically difference with the L5S1 level.

$: there was statically difference with the L4L5 level.

The spinopelvic parameters are shown in Table 3. The lumbar muscle volume and fat fraction varied according to gender (Table 4). The muscle volume was higher in men in all the studied muscles. In contrast, the fat fraction was higher in the multifidus and erector muscles in women.

**Table 3:** Spinopelvic parameters expressed as mean, standard deviation (SD) and range (minimum and maximum).

	Mean/SD	Range		Mean/SD	Range
PT (°)	9.5±7.5	-5–46	SVA (mm)	7.5±20.1	-44–57.6
PI (°)	45.9±9.7	21–70	CPA (°)	8.9±6.0	0–30
SS (°)	36.4±6.6	20–52	SSA (°)	125.3±7.7	109–143
LL: L1-S1 (°)	49.5±11.2	23–73	PI-LL (°)	-3.6±11.2	-34–21
LL: L3-S1(°)	43.8±9.1	18–78	L1S1 (cm)	19.7±2.1	14.78–29
TK (°)	37.3±11.2	10–60	T1S1 (cm)	50.4±3.3	43.7–57.8
TL (°)	11.0±7.7	0–36			

PT: pelvic tilt, PI: pelvic incidence, SS: sacral slope, LL: lumbar lordosis, TK: thoracic kyphosis, TL: thoracolumbar alignment, SVA: sagittal vertical axis, CPA: C2 pelvic angle, SSA: spinosacral angle, PI-LL: lack of lordosis, L1S1: lumbosacral spine length, T1S1: thoracolumbar spine length

**Table 4:** Volumetry and fat fraction expressed as mean and standard deviation (SD) of the lumbar musculature according to gender and side. *: Significant difference for p <0.05.

	Women		Men		
	*Mean*	*SD*	*Mean*	*SD*	*p value*
**Lumbar Multifidus**					
Volumetry Right (mm^3^)	65.6	13.2	81.7	17.7	<0.0001 *
Volumetry Left (mm^3^)	65.6	12.0	80.7	17.7	<0.0001 *
Fat Fraction Right (%)	10.6	3.0	8.7	4.5	<0.0001 *
Fat Fraction Left (%)	11.0	3.1	9.1	4.1	<0.0001 *
**Lumbar Erectors**					
Volumetry Right (mm^3^)	142.2	32.2	191.0	39.5	<0.0001 *
Volumetry Left (mm^3^)	138.7	32.5	188.2	41.2	<0.0001 *
Fat Fraction Right (%)	12.2	4.6	10.8	6.4	0.01 *
Fat Fraction Left (%)	12.7	4.5	11.2	6.4	0.007 *
**Psoas**					
Volumetry Right (mm^3^)	76.5	16.8	131.9	27.6	<0.0001 *
Volumetry Left (mm^3^)	74.7	17.1	130.7	28.4	<0.0001 *
Fat Fraction Right (%)	6.8	2.8	7.6	4.8	0.87
Fat Fraction Left (%)	6.8	2.6	7.7	4.7	0.88

Table 5 presents the correlation values (R) between anthropometric characteristics of our sample (height, weight and BMI). Fat fraction on lumbar multifidus and erectors showed a small correlation with height, weight, and BMI. However, psoas fat fraction was not correlated with any anthropometric characteristics. Multifidus volumes exhibited a moderated correlation with the anthropometric characteristics. Psoas and erectors volumes showed a moderate to strong correlation with height and weight.

**Table 5:** Correlation of anthropometric characteristics with fat fraction and volume values on lumbar musculature. * Denotes a significant correlation (p <0.05).

	**Lumbar Multifidus**			
	*Vol*. *R*	*Vol*. *L*	*FF R*	*FF L*
Height	0.48 *	0.53 *	0.25 *	0.26 *
Weight	0.58 *	0.61 *	0.32 *	0.35 *
BMI	0.46 *	0.46 *	0.25 *	0.28 *
	**Lumbar Erectors**			
	*Vol*. *R*	*Vol*. *L*	*FF R*	*FF L*
Height	0.67 *	0.67 *	0.27 *	0.28 *
Weight	0.71 *	0.72 *	0.28 *	0.31 *
BMI	0.50 *	0.53 *	0.16	0.19
	**Psoas**			
	*Vol*. *R*	*Vol*. *L*	*FF R*	*FF L*
Height	0.72 *	0.74 *	0.02	0.01
Weight	0.68 *	0.71 *	0.12	0.14
BMI	0.47 *	0.50 *	0.14	0.19

Vol. R: Volume muscle right side, Vol. L: volume muscle left side, FF R: fat fraction right side, FF L: fat fraction left side, BMI: Body Mass Index

Table 6 shows the correlations (R) between among the lumbar muscle morphometry, volumetry, FF and spinopelvic parameters. Lumbar multifidus volume was weakly correlated with pelvic tilt, pelvic incidence, lumbar lordosis, CPA and L1S1 length. Multifidus volume also presented a moderate correlation with T1S1 length. Lumbar erector spinae volume was weakly correlated with CPA and had a weak to moderate correlation with L1S1 length and T1S1 length. Psoas volumes were correlated with thoracic kyphosis; TL alignment and SVA. Also, psoas volumes showed a negative weak correlation with SSA and a moderate correlation with L1S1 length and T1S1 length. About fat fraction values, only left lumbar erector presented a negative weak correlation with SVA.

**Table 6:** Correlation (r) of the volume and fat fraction values on lumbar musculature with the spinopelvic parameters. * Denotes a significant correlation (p <0.05).

	Lumbar Multifidus				Lumbar Erectors				Psoas			
	*Vol*. *R*	*Vol*. *L*	*FF R*	*FF L*	*Vol*. *R*	*Vol*. *L*	*FF R*	*FF L*	*Vol*. *R*	*Vol*. *L*	*FF R*	*FF L*
PT (r)	0.22 *	0.16	–0.21	–0.17	0.09	0.08	0.009	–0.002	0.03	0.06	–0.20	–0.14
PI (r)	0.22 *	0.20	–0.11	–0.08	0.004	–0.01	0.03	0.04	–0.03	–0.01	–0.10	–0.02
SS (r)	0.08	0.09	0.15	0.15	–0.05	–0.06	0.07	0.08	–0.12	–0.11	0.07	0.18
LL (r)	0.31 *	0.34 *	0.14	0.14	0.04	0.03	0.18	0.17	0.05	0.06	–0.003	0.10
LL: L1S3(r)	-0.04	-0.01	0.13	0.10	-0.04	-0.05	0.14	0.12	-0.01	-0.04	0.13	0.14
TK (r)	0.18	0.18	0.006	0.01	0.12	0.15	–0.14	–0.14	0.21 *	0.18	0.01	0.06
TL (r)	0.03	0.07	–0.08	–0.14	0.19	0.18	–0.14	–0.15	0.27 *	0.25 *	0.02	–0.01
SVA (r)	0.07	0.06	–0.14	–0.14	0.17	0.18	–0.17	–0.22 *	–0.23 *	–0.21 *	0.007	0.01
CPA (r)	0.29 *	0.24 *	–0.14	–0.10	0.21 *	0.16	0.02	0.01	0.11	0.11	–0.15	–0.11
SSA (r)	0.02	0.04	0.15	0.17	–0.14	–0.16	0.05	0.08	–0.26 *	–0.25 *	0.11	0.15
PI–LL (r)	0.06	0.03	–0.24 8	–0.18	0.04	0.03	–0.16	–0.14	–0.12	0.12	–0.09	–0.11
L1S1 (r)	0.20 *	0.24 *	–0.06	–0.07	0.38 *	0.40 *	–0.05	–0.03	0.41 *	0.40 *	0.02	0.03
T1S1 (r)	0.45 *	0.46 *	–0.14	–0.16	0.56 *	0.55 *	–0.07	–0.06	0.62 *	0.62 *	0.07	0.08

Vol. R: Volume muscle right side, Vol. L: volume muscle left side, FF R: fat fraction right side, FF L: fat fraction left side, PT: pelvic tilt, PI: pelvic incidence, SS: sacral slope, LL: lumbar lordosis, TK: thoracic kyphosis, TL: thoracolumbar alignment, SVA: sagittal vertical axis, CPA: C2 pelvic angle, SSA: spinosacral angle, PI–LL: lack of lordosis, L1S1: lumbosacral spine length, T1S1: thoracolumbar spine length

There was no correlation between most lumbar intervertebral angles and the fat fraction or CSA of the lumbar muscles. However, we found an inverse correlation between L4-L5 intervertebral angle and the right side volumes of psoas (R = -0.27) and lumbar erectors (R = -0.21).

Table 7 also presents the reliability analysis based on the intra- and inter-observer results. Robust ICCs (0.77–0.97) with short confidence intervals were observed.

**Table 7:** Intraclass coefficient (ICC) and confidence interval (95% CI) for interobserver and intraobserver analysis on muscle volumes and spinopelvic parameters.

	Interobserver		Intraobserver	
	*ICC*	*95% CI*	*ICC*	*95% CI*
Left Erector	0.89	0.81–0.91	0.90	0.82–0.90
Right Erector	0.87	0.83–0.90	0.82	0.70–0.87
Left Multifidus	0.86	0.72–0.88	0.88	0.78–0.92
Right Multifidus	0.81	0.79–0.87	0.83	0.80–0.92
Left Psoas	0.83	0.78–0.96	0.89	0.81–0.97
Right Psoas	0.84	0.78–0.91	0.88	0.78–0.89
SS	0.93	0.88–0.96	0.90	0.72–0.97
PT	0.77	0.59–0.87	0.93	0.78–0.98
PI	0.91	0.85–0.96	0.97	0.90–0.99
LL	0.93	0.87–0.96	0.85	0.78–0.95
TK	0.88	0.85–0.97	0.80	0.73–0.93
TL	0.90	0.88–0.96	0.85	0.78–0.96
CPA	0.91	0.87–0.93	0.84	0.73–0.96
SVA	0.89	0.81–0.91	0.90	0.81–0.98
SSA	0.87	0.83–0.90	0.89	0.80–0.90
PI–LL	0.91	0.85–0.87	0.85	0.78–0.95
T1S1	0.93	0.90–0.96	0.92	0.87–0.99
L1S1	0.90	0.89–0.95	0.94	0.89–0.99

## Discussion

In this research study, we investigated the relationships among the spinopelvic parameters, morphology and fat fraction of the lumbar paraspinal musculature. We found correlations between the lumbar multifidus volumes and LL, PT, PI and CPA and between the lumbar erectors and CPA; the psoas volume correlated with the TK, TL, and SVA and was inversely related to SSA. All correlations were weak (R = 0.20–0.34). All muscle volumes correlated moderately with the spine length (T1S1 and L1S1 length). Regarding the muscle fat fraction, only the lumbar erectors showed a weak inverse correlation with the SVA.

We found muscle asymmetry between the right and left sides in the healthy volunteers (Table 1). These findings have been previously reported in the literature. Niemelainem et al. [22] reported that the cross-sectional area asymmetry in the erector muscles in a healthy male sample was 40%. Valentin et al. [23] observed muscle asymmetry in older adults and healthy subjects 45–60 years of age by assessing the volume of the lumbar erector and rectus abdominal muscles. Marras et al.[24] identified asymmetry in the psoas muscle in a population of young healthy adults. Our results confirm that paraspinal muscle asymmetries may be present in a population of asymptomatic young adults.

In our study, the lumbar muscle volumes in men were greater than those in women, and the multifidus and erector spinae fat fraction in women was higher than that in men (Table 4). Kjaer et al. [25] found that women had a higher fat infiltration in the lumbar multifidus that men by studying a population of 412 adults and 442 adolescents with LBP. Sions et al. [26] observed that in an older population, women had smaller multifidi, erector spinae, psoas, and quadratus CSAs than men at every vertebral level. Additionally, these authors reported that females had an increased amount of intramuscular fat in the trunk muscles at the lower vertebral levels. In our study, psoas FF showed no differences between genders. Urrutia et al. [27] showed an association between sex and age with psoas fat infiltration. However, those authors evaluated only symptomatic and elderly subjects. Our volunteer's age range 20–40 years and our results suggest that differences in psoas muscle between genders are not evident in young adults.

Table 2 shows that there was a significant craniocaudal increase in the fat fraction for multifidus and erector muscles, but the psoas fat fraction at the L4-L5 level was higher than L5-S1. Extensor muscles at the L5-S1 level tended to have smaller CSAs. Crawford et al. [2] results are in agreement with our results about muscle volume and fat fraction in the multifidus and erectors. Our results are also partially in agreement with the age- and level-dependence of paravertebral muscle fatty infiltration identified by Lee et al. [28]. These authors showed increased fatty infiltration of the multifidus and erector spinae muscles at the lower lumbar levels, on asymptomatic young, middle and elderly population. According to previous reports, spine degeneration and muscle fatty infiltration have mutual effects on each other [28]. Lee et al found no age or level dependency for psoas muscle fat infiltration, on the other hand, with minimal muscle fatty infiltration. In our study, the psoas muscle showed higher FF at the L4-L5 level than in other levels. We could not elaborate any speculation about why psoas showed higher FF at L4-L5.

The muscle volumetry showed a moderated correlation with anthropometric characteristics (height, weight, and BMI), while the fat fraction of multifidus and erector muscle showed weak correlation with the same parameters. Crawford et al. describe no association between BMI and the paraspinal fat fraction on asymptomatic volunteers of both genders [2]. On the other hand, Fortin et al. [29] performed a 15 years longitudinal follow-up and identified a great relationship between the increase in BMI and the presence of fatty infiltrate in the paraspinal muscles. However, Fortin et al. studied a male-based population. Consideration should be given to sexual characteristics differences in relation to muscle mass and body fat concentration. These authors confirm that such longitudinal findings corroborate with other previously described cross-sectional studies of increased body mass accompanied by an increase in intramuscular fat infiltration.

Meakin et al. [14] found a moderate correlation (R = 0.61) between the lumbar extensor muscle volumes and lordosis, whereas we found a weak correlation between the LL and the multifidus volumes. The apparent discrepancy between our results and those reported by Meakin et al. [14] may be due to differences in the methodologies. They measured L4-S1 lordosis, while we measured L1-S1 lordosis. Additionally, Meakin et al. measured the muscle volumes only at the L3 to L4 levels, while we measured lumbar muscles volumes from L3 to S1. When we measured lordosis from L3 to S1 we found no significant correlation with lumbar muscle volumes (Table 3). Maybe that the shape characteristics of the upper versus lower lumbar spine are also an important influencing factor. Likewise, we found no correlation between most intervertebral angles and muscular CSA at the same levels (L3-L4, L4-L5, and L5-S1). However, the L4-L5 intervertebral angle had a weak negative correlation with the volume of right psoas (R = -0.27) and right erectors (R = -0.21). The L4-L5 segment had the highest frequencies of a clinically significant lumbar disc and facet joint degeneration [30] and its intervertebral angle presents the widest angular variation between different spinal alignment types [31]. Spines with flat lordosis exhibited a higher frequency of L4-L5 disc desiccation than spines with greater lordosis on asymptomatic young adults [16]. This higher tendency for degeneration at the L4-L5 level may explain these results at least in part, considering the relationship between stress loading and muscle fatty degeneration [28]. Future studies focused on paraspinal muscle quality and lumbar spine alignment relationship on elderly or symptomatic patients are encouraged.

Jun et al. [32] identified a weak correlation between the LL and lumbar erector muscles (R = 0.34) in an elderly population. Interestingly, in our sample, the lumbar erectors were not correlated with LL, but we found a significant correlation when we analyzed the multifidus separately.

PI and PT showed weak positive correlations with the multifidus volumes. To the best of our knowledge, there have been no previous investigations of the correlations between PI, PT or SS and muscle morphology in young adults. PI is a fixed parameter that does not change with movement or postural changes. The pelvic angle parameters are strong predictors of the magnitude of lordosis [33]. Because of the interdependency between PI and LL, the correlation between the multifidus volume and both PI and LL is logical. However, PT is a dynamic pelvic parameter that increases or decreases due to rotations of the hip axis, and it can change with different postures. PT increases as a compensatory mechanism, which requires both effort and energy to maintain a global alignment [11], which is consistent with our results in which slight increases in the multifidus volume were observed with higher PT values.

CPA correlated with both the multifidus and erector volumes. This angle reflects the global sagittal balance, which is influenced by pelvic retroversion and sagittal curvatures similarly to SSA. To the best of our knowledge, no previous studies have correlated global spinopelvic parameters with the paraspinal muscles.

The psoas volume increased slightly as the TK angle and TL increased. Additionally, the thoracolumbar angle (TL angle) was positively correlated with the psoas muscle volume. The psoas muscle originates from the transverse processes of T12-L5 [34]; therefore, an increased transitional thoracolumbar curve may be involved in the increased psoas activation. In contrast, Masaki et al. [35] did not find a relationship between TK and psoas thickness using ultrasonography. In addition to the methodological differences, those authors measured psoas thickness with ultrasonography, while we measured psoas muscle volume using MRI; their study included only middle-aged and elderly women, whereas we studied young asymptomatic adults.

SVA was inversely correlated with the psoas volume. According to a recent review [36], positive SVA indicates a progressive anterior translation of the head away from the pelvis and provides information regarding the general trunk alignment. Lower psoas fascicles are responsible for flexing the lumbar spine [37]. Based on our results, we hypothesize that subjects with negative SVA have a greater psoas activation, explaining the higher muscle volume.

In our study, the multifidus, erectors, and psoas muscle volumes presented weak to moderate correlations with the thoracolumbar and lumbar spinal length (Table 6). The T1S1 length showed higher correlation values than the L1S1 length. This result is in agreement with our expectation since muscle volumetry also showed a good correlation with the volunteer's height (Table 4). Interestingly the literature tends to point to a higher influence of spinal curvatures than spinal length on muscle trophism. A previous longitudinal study may help to understand the correlation between paraspinal muscle changes, especially on lumbar multifidus and spine length [38]. Therefore spinal length may influence muscle composition, or vice-versa.

Regarding the fat infiltration, we found a weak negative correlation between SVA and the lumbar erector spinae. As previously described, the SVA may indicate an anterior spinal imbalance [39]. These characteristics are commonly found in subjects with LBP who exhibit higher values of fat infiltration than healthy people [6,32,40]. No other parameters in our study showed correlations with the fat fraction in any muscle. In the literature, the relationship between paraspinal musculature fat infiltration and spinal diseases is already well-established [10,32]. The reason we did not find a significant correlation between fat infiltration and the sagittal plane parameters probably occurred because we investigated healthy individuals without a sagittal plane imbalance.

Crawford et al. [36] showed [2,28] that the annual rate of increasing paravertebral muscle fat content was low, and suggests a relatively slow decline in lumbar muscle quality into healthy adulthood. Jun et al. found a relationship between muscular composition and some spinopelvic parameters in the elderly population [32]. We believe that our correlations were not as pronounced because our volunteers are young (age 20–40). Our results indicate that other factors different from spinopelvic parameters may exert greater influence on lumbar muscles trophism on young adults.

It is important to point that there is a great morphological variability of the paraspinal muscles in front of several factors. Fortin et al. found that paraspinal muscle size or fat infiltration asymmetries were not associated with symptoms duration, but are present at the same level of herniated discs [3]. However, another study suggests the association of multifidus muscle fat infiltration and psoas muscle size with the functional status in patients diagnosed with lumbar spinal stenosis [4]. Crawford et al. also suggest that the rate of change of lumbar muscle composition may differ between ethnicities [41]. In addition, some studies performed in extreme situations such as gravity reduction showed intense effects on atrophy and fat infiltration in the paraspinal muscles [7,38]. Futures studies are necessary for better understanding of mechanisms with influence on paraspinal muscle composition.

There were some limitations in our study. First, our study used a cross-sectional design, and thus, it is impossible to infer cause and effect relationships between the variables. The MRI evaluation comprised the levels of L3 to S1. Therefore, the upper lumbar musculature was not evaluated. The statistical correlations obtained were mostly weak to moderate meaning that other factors should account for the variation on paravertebral muscle volume. We evaluated muscle morphological findings using MRI but we do not evaluate muscle activity with electromyography, and such information could be potentially valuable. Additionally, we studied volunteers with low levels of physical activity to avoid the possible influence of athletic activities on the muscle quality. However, investigating the effects of these variables on subjects with high athletic performance would be interesting.

We conclude that the spinopelvic parameters have a significant but weak correlation with the muscle volumes of the lumbar multifidus, erector spinae and psoas in asymptomatic young adults. The lumbar multifidus, erector spinae, and psoas muscle volumes had a moderate correlation with the thoracolumbar spine length. Fat infiltration of the lumbar muscles showed no significant correlations with the spinopelvic parameters in asymptomatic young adults.

## Supporting information

S1 Table Raw data: Spinopelvic parameters and muscle composition (volume and fat infiltration) of lumbar multifidus, lumbar erectors and psoas. (DOCX)
