# Supplementary material for: Relationship of spinal alignment with muscular volume and fat infiltration of lumbar trunk muscles
Source: PLoS One. 2018 Jul 5;13(7):e0200198. doi: 10.1371/journal.pone.0200198 (PMC6033454; doi:10.1371/journal.pone.0200198)
Supplement: S1 Table — (DOCX) [file pone.0200198.s001.docx]

**S1 Table**. **Raw data:** Spinopelvic parameters and muscle composition (volume and fat infiltration) of lumbar multifidus, lumbar erectors and psoas. PT: pelvic tilt, PI: pelvic incidence, SS: sacral slope, LL: lumbar lordosis, TK: thoracic kyphosis, TL: thoracolumbar alignment, SVA: sagittal vertical axis, CPA: C2 pelvic angle, SSA: spinosacral angle, PI-LL: lack of lordosis, L1S1: lumbosacral spine length, T1S1: thoracolumbar spine length, Vol. R: Volume muscle right side, Vol. L: volume muscle left side, FF R: fat fraction right side, FF L: fat fraction left side, m: male, f: female.

|  |  |  |  |  |  |  |  |  |  |  |  |  |  | **MULTIFIDUS** | | | | **ERECTORS** | | | | **PSOAS** | | | |
| --- | --- | --- | --- | --- | --- | --- | --- | --- | --- | --- | --- | --- | --- | --- | --- | --- | --- | --- | --- | --- | --- | --- | --- | --- | --- |
| Volunteer | Genre | PT | PI | SS | LL | TL | TK | SVA | CPA | L1-S1 | T1-S1 | SSA | PI-LL | Vol_R | Vol_L | Fat R | Fat L | Vol_R | Vol_L | Fat R | Fat L | Vol_R | Vol_L | Fat R | Fat L |
| 1 | m | 5 | 39 | 34 | 56 | 16 | 46 | -11,0 | 5 | 20,1 | 53,8 | 124 | -17 | 80,0 | 75,4 | 23,6 | 19,5 | 187,9 | 179,8 | 19,8 | 20,6 | 126,3 | 126,1 | 16,8 | 18,6 |
| 2 | f | 7 | 42 | 34 | 36 | 14 | 43 | -8,1 | 5 | 17,8 | 50,0 | 126 | 6 | 87,4 | 89,5 | 9,9 | 9,8 | 112,5 | 106,3 | 10,6 | 10,2 | 81,7 | 77,8 | 6,5 | 7,6 |
| 3 | f | 5 | 49 | 44 | 56 | 8 | 37 | 26,9 | 6 | 19,7 | 50,7 | 128 | -7 | 70,1 | 67,4 | 10,9 | 10,3 | 148,1 | 139,2 | 11,8 | 11,4 | 92,3 | 89,4 | 5,8 | 5,6 |
| 4 | f | 24 | 59 | 35 | 42 | 6 | 12 | 7,5 | 10 | 18,5 | 47,1 | 121 | 17 | 60,4 | 60,2 | 10,0 | 10,7 | 117,0 | 115,8 | 19,4 | 20,1 | 71,7 | 67,8 | 6,5 | 7,1 |
| 5 | f | 1 | 44 | 43 | 47 | 5 | 45 | -31,8 | 0 | 19,3 | 46,1 | 139 | -3 | 77,7 | 82,1 | 11,4 | 11,2 | 159,9 | 163,0 | 10,9 | 11,5 | 79,5 | 73,2 | 6,8 | 6,6 |
| 6 | m | 12 | 59 | 46 | 62 | 9 | 38 | 0,3 | 11 | 20,0 | 53,4 | 136 | -3 | 109,1 | 106,1 | 9,0 | 10,9 | 167,0 | 147,9 | 10,2 | 11,6 | 126,0 | 121,1 | 7,0 | 6,4 |
| 7 | f | 4 | 43 | 38 | 40 | 3 | 25 | 19,3 | 17 | 21,1 | 53,1 | 119 | 3 | 42,1 | 47,3 | 8,6 | 9,6 | 142,1 | 130,8 | 18,0 | 19,8 | 86,9 | 80,8 | 3,7 | 4,1 |
| 8 | f | 8 | 44 | 36 | 39 | 3 | 21 | -10,9 | 2 | 18,1 | 48,3 | 126 | 5 | 54,7 | 57,1 | 10,2 | 10,1 | 92,3 | 96,9 | 9,8 | 9,8 | 58,6 | 58,3 | 6,0 | 5,0 |
| 9 | f | 12 | 46 | 34 | 41 | 0 | 35 | 26,9 | 14 | 19,5 | 50,6 | 121 | 5 | 79,5 | 72,9 | 13,6 | 13,8 | 153,2 | 152,5 | 14,5 | 14,9 | 74,6 | 65,2 | 9,2 | 8,0 |
| 10 | m | 13 | 46 | 33 | 63 | 14 | 60 | 23,6 | 12 | 17,1 | 46,6 | 120 | -17 | 74,0 | 66,0 | 5,5 | 6,6 | 135,8 | 123,3 | 6,3 | 6,4 | 96,4 | 96,2 | 4,7 | 4,5 |
| 11 | f | 1 | 31 | 30 | 44 | 27 | 40 | -16,4 | 3 | 19,1 | 47,9 | 124 | -13 | 74,6 | 76,8 | 9,1 | 10,0 | 175,7 | 178,1 | 8,8 | 10,4 | 103,3 | 96,9 | 5,3 | 5,2 |
| 12 | f | 10 | 59 | 49 | 73 | 6 | 31 | -20,0 | 6 | 19,5 | 50,9 | 143 | -14 | 87,9 | 86,1 | 12,7 | 11,2 | 186,9 | 193,5 | 24,1 | 23,1 | 119,6 | 117,6 | 7,0 | 8,2 |
| 13 | f | 18 | 55 | 38 | 58 | 8 | 43 | -9,8 | 14 | 26,4 | 44,4 | 128 | -3 | 61,0 | 66,3 | 8,1 | 9,6 | 94,4 | 95,8 | 7,8 | 8,6 | 79,2 | 71,2 | 3,8 | 4,4 |
| 14 | f | 16 | 63 | 47 | 48 | 8 | 41 | 10,6 | 16 | 19,6 | 50,5 | 139 | 15 | 79,3 | 75,5 | 8,2 | 8,3 | 146,8 | 131,5 | 11,6 | 13,2 | 79,3 | 75,5 | 8,2 | 8,3 |
| 15 | m | 17 | 50 | 33 | 47 | 21 | 32 | 6,8 | 14 | 19,1 | 49,2 | 112 | 3 | 69,0 | 68,5 | 9,4 | 9,3 | 156,9 | 152,9 | 7,9 | 8,1 | 126,2 | 128,6 | 4,6 | 4,1 |
| 16 | f | 9 | 54 | 45 | 67 | 6 | 42 | 12,5 | 10 | 19,0 | 48,5 | 138 | -13 | 69,2 | 66,0 | 12,5 | 14,4 | 129,4 | 122,5 | 12,9 | 14,5 | 63,3 | 59,3 | 10,0 | 9,8 |
| 17 | m | 14 | 55 | 41 | 60 | 11 | 49 | 16,2 | 8 | 20,6 | 54,4 | 123 | -5 | 75,2 | 74,9 | 12,1 | 12,7 | 157,4 | 158,5 | 11,3 | 11,8 | 91,9 | 99,1 | 8,2 | 8,0 |
| 18 | f | 11 | 48 | 37 | 52 | 15 | 29 | 18,4 | 11 | 18,3 | 47,5 | 128 | -4 | 87,1 | 81,9 | 8,4 | 9,3 | 146,5 | 135,9 | 17,8 | 18,6 | 89,9 | 84,7 | 5,4 | 5,7 |
| 19 | m | 15 | 60 | 44 | 39 | 11 | 16 | 35,3 | 11 | 19,3 | 50,8 | 132 | 21 | 52,0 | 51,7 | 2,4 | 2,3 | 190,7 | 177,7 | 1,3 | 1,0 | 111,5 | 114,4 | 3,3 | 2,6 |
| 20 | m | 2 | 43 | 41 | 63 | 20 | 46 | 13,3 | 2 | 19,8 | 50,7 | 125 | -20 | 67,5 | 67,1 | 8,4 | 10,2 | 154,1 | 152,5 | 18,5 | 19,8 | 140,7 | 129,3 | 5,3 | 5,8 |
| 21 | m | 10 | 45 | 35 | 47 | 12 | 36 | 27,4 | 12 | 21,0 | 54,2 | 121 | -2 | 88,6 | 80,2 | 5,6 | 5,8 | 217,0 | 201,6 | 5,9 | 5,9 | 92,0 | 96,6 | 6,4 | 6,2 |
| 22 | m | 1 | 42 | 40 | 64 | 15 | 49 | -2,3 | 1 | 19,1 | 49,0 | 129 | -22 | 66,4 | 65,1 | 13,5 | 13,8 | 128,3 | 121,1 | 13,8 | 15,4 | 78,1 | 72,1 | 13,6 | 11,7 |
| 23 | f | 4 | 47 | 43 | 48 | 5 | 33 | 33,0 | 5 | 19,8 | 51,6 | 128 | -1 | 64,9 | 58,9 | 11,9 | 12,4 | 122,0 | 115,9 | 12,6 | 13,2 | 65,5 | 68,9 | 8,7 | 9,2 |
| 24 | f | 10 | 50 | 39 | 66 | 18 | 50 | 14,6 | 10 | 18,0 | 43,7 | 128 | -16 | 65,1 | 67,7 | 13,0 | 12,5 | 150,2 | 150,5 | 12,4 | 11,7 | 74,9 | 68,1 | 5,7 | 5,9 |
| 25 | f | 11 | 55 | 44 | 67 | 0 | 41 | -8,3 | 8 | 18,9 | 48,8 | 134 | -12 | 85,7 | 91,7 | 8,3 | 8,8 | 189,9 | 188,9 | 6,9 | 7,3 | 82,9 | 87,1 | 5,1 | 5,1 |
| 26 | f | 0 | 42 | 42 | 48 | 17 | 40 | -24,4 | 6 | 21,8 | 55,8 | 135 | -6 | 85,2 | 81,7 | 10,7 | 11,3 | 193,7 | 193,4 | 11,0 | 11,6 | 85,0 | 90,3 | 7,4 | 7,0 |
| 27 | f | 4 | 50 | 45 | 54 | 13 | 55 | -22,4 | 4 | 16,9 | 44,5 | 140 | -4 | 46,1 | 48,0 | 10,6 | 11,2 | 101,3 | 107,5 | 10,1 | 11,2 | 58,2 | 60,1 | 7,8 | 8,6 |
| 28 | f | -2 | 33 | 35 | 59 | 7 | 40 | -44,2 | 0 | 19,0 | 46,7 | 131 | -26 | 52,6 | 53,3 | 8,9 | 9,9 | 108,2 | 106,6 | 9,7 | 11,6 | 82,8 | 79,2 | 7,3 | 7,3 |
| 29 | m | 22 | 58 | 36 | 53 | 9 | 20 | -11,6 | 19 | 19,5 | 55,6 | 128 | 5 | 101,5 | 98,8 | 6,1 | 8,3 | 169,0 | 160,0 | 19,6 | 21,3 | 165,5 | 160,0 | 5,7 | 7,1 |
| 30 | m | 7 | 56 | 49 | 68 | 0 | 43 | -9,5 | 4 | 21,9 | 57,8 | 139 | -12 | 98,3 | 96,5 | 7,2 | 8,0 | 192,0 | 186,1 | 8,7 | 9,6 | 138,2 | 137,6 | 8,8 | 7,6 |
| 31 | m | 13 | 54 | 40 | 57 | 0 | 44 | -1,5 | 11 | 14,8 | 51,3 | 130 | -3 | 85,7 | 79,9 | 6,3 | 6,5 | 167,7 | 173,4 | 26,7 | 25,9 | 113,8 | 112,4 | 5,7 | 7,1 |
| 32 | m | 19 | 65 | 45 | 62 | 8 | 51 | 2,1 | 12 | 20,3 | 54,1 | 132 | 3 | 98,0 | 109,3 | 8,4 | 9,7 | 231,9 | 217,7 | 9,5 | 9,5 | 193,9 | 186,9 | 3,6 | 4,7 |
| 33 | m | 6 | 29 | 23 | 37 | 31 | 42 | 6,0 | 2 | 19,8 | 52,6 | 113 | -8 | 101,1 | 105,1 | 8,8 | 9,8 | 282,8 | 281,6 | 10,5 | 11,1 | 163,2 | 176,5 | 6,9 | 7,1 |
| 34 | m | 9 | 42 | 33 | 47 | 30 | 21 | 3,7 | 8 | 21,1 | 54,6 | 112 | -5 | 80,8 | 75,8 | 6,9 | 7,4 | 215,8 | 213,5 | 22,7 | 23,0 | 131,2 | 128,5 | 6,0 | 6,8 |
| 35 | m | 10 | 40 | 30 | 50 | 25 | 49 | 17,9 | 9 | 22,3 | 53,7 | 115 | -10 | 76,5 | 71,8 | 7,1 | 8,1 | 221,3 | 207,4 | 12,1 | 12,8 | 117,0 | 120,5 | 5,8 | 5,7 |
| 36 | m | 15 | 56 | 41 | 61 | 18 | 60 | 57,6 | 15 | 19,8 | 50,8 | 127 | -5 | 87,6 | 87,0 | 6,2 | 6,5 | 181,3 | 178,8 | 7,5 | 7,9 | 143,5 | 141,3 | 5,7 | 8,2 |
| 37 | f | -1 | 23 | 24 | 37 | 23 | 25 | -13,7 | 2 | 19,6 | 49,0 | 119 | -14 | 60,1 | 61,7 | 11,6 | 12,7 | 124,7 | 124,0 | 12,5 | 13,0 | 89,4 | 85,3 | 7,7 | 7,0 |
| 38 | m | 46 | 60 | 43 | 55 | 4 | 47 | 15,1 | 15 | 20,6 | 52,8 | 130 | 5 | 77,7 | 78,9 | 7,0 | 8,2 | 155,1 | 154,4 | 7,6 | 9,0 | 119,1 | 119,2 | 5,5 | 6,5 |
| 39 | f | 3 | 29 | 26 | 39 | 22 | 28 | 22,3 | 1 | 18,4 | 47,8 | 123 | -10 | 64,4 | 71,7 | 11,4 | 10,7 | 154,1 | 124,3 | 10,4 | 10,6 | 64,3 | 68,2 | 8,5 | 8,3 |
| 40 | m | 11 | 46 | 35 | 49 | 7 | 33 | 10,8 | 10 | 18,5 | 46,1 | 128 | -3 | 52,9 | 52,4 | 17,2 | 17,3 | 128,4 | 120,2 | 16,8 | 17,9 | 109,3 | 116,5 | 16,9 | 17,3 |
| 41 | m | 18 | 57 | 38 | 41 | 15 | 40 | 32,6 | 30 | 20,3 | 55,5 | 125 | 16 | 66,1 | 72,4 | 6,6 | 7,1 | 182,2 | 195,6 | 8,2 | 8,5 | 108,5 | 101,8 | 6,9 | 7,0 |
| 42 | f | 9 | 48 | 38 | 45 | 11 | 44 | 31,4 | 12 | 20,1 | 51,2 | 123 | 3 | 64,3 | 70,9 | 11,3 | 11,4 | 126,6 | 142,2 | 11,3 | 11,5 | 117,0 | 98,5 | 7,7 | 7,3 |
| 43 | f | 17 | 55 | 37 | 48 | 1 | 35 | 25,7 | 12 | 18,0 | 47,6 | 130 | 7 | 75,0 | 74,0 | 0,0 | 0,1 | 137,9 | 137,3 | 0,0 | 0,1 | 72,9 | 69,8 | 0,1 | 0,7 |
| 44 | f | 6 | 34 | 27 | 42 | 17 | 37 | 11,7 | 12 | 20,4 | 51,4 | 120 | -8 | 70,2 | 69,0 | 13,0 | 13,2 | 196,5 | 201,5 | 11,1 | 12,2 | 82,3 | 90,7 | 7,7 | 6,2 |
| 45 | f | 8 | 53 | 46 | 61 | 6 | 27 | 13,3 | 9 | 29,0 | 47,5 | 132 | -8 | 68,2 | 66,0 | 12,7 | 11,5 | 113,9 | 110,6 | 10,9 | 10,0 | 57,5 | 60,7 | 5,7 | 6,6 |
| 46 | m | 6 | 45 | 39 | 54 | 15 | 45 | 27,6 | 5 | 19,6 | 51,8 | 125 | -9 | 75,6 | 73,6 | 7,9 | 8,0 | 182,0 | 181,0 | 9,2 | 8,8 | 128,2 | 126,0 | 6,8 | 6,9 |
| 47 | f | 5 | 49 | 43 | 49 | 3 | 30 | 9,9 | 3 | 17,8 | 46,0 | 135 | 0 | 55,2 | 59,4 | 10,1 | 12,4 | 154,5 | 160,0 | 9,9 | 10,5 | 63,1 | 70,5 | 6,8 | 8,0 |
| 48 | f | 14 | 51 | 37 | 60 | 3 | 22 | -20,5 | 10 | 18,5 | 48,3 | 121 | -9 | 66,3 | 64,8 | 8,5 | 8,5 | 114,3 | 111,5 | 17,3 | 17,7 | 66,7 | 65,6 | 4,9 | 5,1 |
| 49 | f | -4 | 36 | 40 | 61 | 1 | 50 | 9,7 | 2 | 18,3 | 46,0 | 132 | -25 | 33,8 | 43,0 | 10,4 | 11,8 | 96,7 | 101,6 | 7,5 | 9,3 | 33,2 | 27,5 | 4,8 | 6,4 |
| 50 | m | 11 | 35 | 24 | 37 | 11 | 31 | 38,8 | 13 | 19,0 | 51,8 | 109 | -2 | 82,3 | 79,3 | 5,4 | 5,3 | 182,8 | 178,5 | 5,5 | 5,7 | 116,0 | 133,1 | 5,9 | 6,0 |
| 51 | f | 3 | 41 | 39 | 54 | 6 | 23 | 12,0 | 1 | 18,4 | 47,8 | 129 | -13 | 53,5 | 53,7 | 19,0 | 20,1 | 128,4 | 121,0 | 19,0 | 20,4 | 81,7 | 81,3 | 15,8 | 15,4 |
| 52 | f | 5 | 38 | 33 | 39 | 16 | 33 | -12,5 | 5 | 20,9 | 54,1 | 124 | -1 | 44,6 | 52,4 | 8,0 | 8,5 | 161,7 | 140,6 | 5,3 | 6,2 | 55,3 | 52,1 | 4,0 | 5,3 |
| 53 | m | 9 | 47 | 37 | 61 | 6 | 50 | 20,4 | 17 | 17,3 | 51,5 | 123 | -14 | 105,1 | 101,1 | 12,0 | 11,8 | 214,5 | 209,9 | 12,3 | 11,0 | 144,7 | 161,3 | 8,2 | 7,9 |
| 54 | m | 10 | 39 | 29 | 29 | 2 | 27 | 43,3 | 15 | 20,5 | 53,7 | 116 | 10 | 80,2 | 76,7 | 6,4 | 8,5 | 199,8 | 201,4 | 7,1 | 8,0 | 136,9 | 132,0 | 8,0 | 8,2 |
| 55 | m | 15 | 53 | 38 | 52 | 6 | 19 | 20,6 | 14 | 20,4 | 53,4 | 131 | 1 | 69,1 | 73,2 | 14,5 | 13,9 | 167,2 | 177,0 | 13,9 | 13,9 | 125,9 | 125,8 | 15,5 | 16,2 |
| 56 | m | 10 | 47 | 36 | 51 | 5 | 33 | 30,1 | 4 | 21,8 | 56,0 | 118 | -4 | 71,9 | 72,2 | 9,1 | 8,1 | 178,2 | 185,4 | 8,1 | 8,0 | 141,8 | 138,1 | 6,3 | 5,8 |
| 57 | m | 0 | 28 | 28 | 46 | 14 | 40 | 4,2 | 1 | 19,1 | 49,1 | 118 | -18 | 50,3 | 47,1 | 5,7 | 4,5 | 182,7 | 175,4 | 4,4 | 3,6 | 143,9 | 133,7 | 4,3 | 4,8 |
| 58 | f | 7 | 34 | 26 | 45 | 24 | 43 | 5,9 | 2 | 18,1 | 46,3 | 113 | -11 | 60,7 | 63,2 | 11,5 | 9,0 | 120,5 | 124,1 | 11,1 | 9,9 | 79,2 | 68,0 | 8,0 | 7,6 |
| 59 | m | -5 | 21 | 26 | 53 | 36 | 60 | 7,7 | 6 | 23,1 | 57,8 | 111 | -32 | 76,9 | 74,6 | 18,8 | 18,0 | 169,5 | 172,0 | 23,8 | 22,9 | 129,2 | 123,0 | 22,4 | 21,6 |
| 60 | m | 0 | 38 | 38 | 50 | 5 | 48 | -5,2 | 1 | 19,9 | 50,4 | 127 | -12 | 71,4 | 70,4 | 10,8 | 10,0 | 172,0 | 172,6 | 10,8 | 10,2 | 107,5 | 100,0 | 8,3 | 7,9 |
| 61 | m | 8 | 32 | 24 | 37 | 18 | 41 | 8,8 | 2 | 21,0 | 54,1 | 111 | -5 | 69,1 | 64,7 | 7,7 | 7,6 | 162,0 | 149,4 | 7,2 | 8,2 | 125,4 | 130,2 | 8,2 | 7,3 |
| 62 | f | 16 | 37 | 20 | 33 | 6 | 49 | -6,8 | 5 | 19,0 | 45,0 | 112 | 4 | 78,2 | 69,7 | 9,9 | 11,9 | 165,7 | 163,1 | 12,8 | 14,1 | 82,6 | 75,6 | 7,6 | 7,3 |
| 63 | f | 21 | 49 | 29 | 54 | 16 | 39 | -30,3 | 15 | 28,6 | 48,5 | 123 | -5 | 54,4 | 53,5 | 10,9 | 10,2 | 142,9 | 145,0 | 12,3 | 12,3 | 71,6 | 71,5 | 4,8 | 6,0 |
| 64 | f | 10 | 48 | 38 | 47 | 15 | 18 | 18,3 | 14 | 17,9 | 45,5 | 130 | 1 | 62,7 | 62,4 | 8,7 | 7,8 | 126,3 | 122,1 | 8,7 | 8,9 | 55,7 | 54,5 | 6,7 | 6,1 |
| 65 | f | 15 | 46 | 31 | 58 | 10 | 48 | -11,7 | 10 | 18,2 | 46,8 | 122 | -12 | 50,9 | 38,8 | 7,4 | 8,7 | 111,7 | 92,5 | 8,4 | 9,3 | 46,5 | 41,2 | 2,8 | 2,9 |
| 66 | m | 0 | 39 | 38 | 29 | 16 | 23 | 52,5 | 9 | 20,8 | 54,8 | 121 | 10 | 76,5 | 75,2 | 6,9 | 7,7 | 148,1 | 169,6 | 6,4 | 7,3 | 134,7 | 118,6 | 5,2 | 6,2 |
| 67 | m | 15 | 46 | 31 | 47 | 19 | 52 | 32,3 | 13 | 21,2 | 55,2 | 119 | -1 | 60,8 | 64,0 | 7,6 | 7,5 | 232,7 | 224,0 | 7,0 | 7,5 | 116,1 | 109,7 | 5,6 | 5,8 |
| 68 | m | 8 | 49 | 41 | 58 | 6 | 45 | 25,1 | 10 | 17,7 | 49,0 | 127 | -9 | 91,6 | 93,3 | 9,2 | 9,9 | 187,9 | 178,4 | 9,4 | 10,0 | 113,9 | 117,8 | 6,4 | 5,6 |
| 69 | m | 15 | 50 | 35 | 52 | 1 | 37 | 36,0 | 13 | 18,4 | 47,1 | 123 | -2 | 72,5 | 64,1 | 2,2 | 3,0 | 167,6 | 152,3 | 12,6 | 13,0 | 97,6 | 93,9 | 3,0 | 3,6 |
| 70 | f | -2 | 29 | 32 | 26 | 8 | 37 | 17,0 | 4 | 19,8 | 49,1 | 119 | 3 | 60,0 | 58,1 | 14,5 | 14,2 | 121,3 | 116,8 | 13,4 | 13,8 | 77,1 | 74,2 | 4,8 | 4,7 |
| 71 | m | 13 | 44 | 31 | 38 | 8 | 32 | 37,4 | 15 | 21,8 | 49,9 | 116 | 6 | 105,1 | 114,4 | 22,1 | 22,5 | 275,6 | 287,1 | 30,1 | 30,3 | 176,6 | 172,8 | 24,5 | 23,9 |
| 72 | f | 8 | 39 | 31 | 37 | 4 | 16 | -14,3 | 8 | 18,7 | 46,9 | 124 | 2 | 44,8 | 43,7 | 8,8 | 8,8 | 113,5 | 103,9 | 18,7 | 19,1 | 59,9 | 54,3 | 4,9 | 3,8 |
| 73 | f | 9 | 48 | 38 | 34 | 13 | 39 | 2,0 | 9 | 19,2 | 48,6 | 126 | 14 | 56,8 | 54,2 | 10,3 | 10,9 | 123,5 | 116,9 | 10,4 | 11,1 | 69,9 | 69,0 | 8,2 | 6,4 |
| 74 | f | 17 | 54 | 37 | 43 | 1 | 40 | 6,4 | 11 | 20,1 | 51,4 | 128 | 11 | 73,4 | 68,8 | 12,0 | 13,4 | 140,1 | 133,9 | 12,8 | 13,4 | 73,2 | 78,6 | 4,6 | 4,3 |
| 75 | f | 16 | 58 | 43 | 64 | 14 | 40 | 13,4 | 14 | 19,5 | 49,9 | 130 | -6 | 76,3 | 75,1 | 10,9 | 12,4 | 187,5 | 179,1 | 11,7 | 12,2 | 79,3 | 75,1 | 6,6 | 7,0 |
| 76 | f | 12 | 52 | 40 | 47 | 11 | 34 | -27,3 | 7 | 19,8 | 51,2 | 130 | 5 | 76,4 | 69,5 | 11,9 | 12,1 | 161,9 | 152,0 | 12,4 | 13,3 | 77,9 | 81,3 | 8,3 | 9,5 |
| 77 | f | 13 | 47 | 33 | 40 | 6 | 33 | 2,1 | 2 | 17,4 | 45,8 | 121 | 7 | 58,6 | 63,9 | 11,5 | 11,4 | 120,8 | 124,8 | 12,6 | 13,4 | 73,4 | 76,0 | 6,0 | 6,8 |
| 78 | f | 5 | 42 | 37 | 23 | 3 | 10 | 14,6 | 11 | 19,3 | 50,2 | 126 | 19 | 76,0 | 75,3 | 8,4 | 9,8 | 258,0 | 258,4 | 8,9 | 9,6 | 71,3 | 62,7 | 5,6 | 5,4 |
| 79 | f | 25 | 70 | 44 | 63 | 2 | 25 | 11,6 | 27 | 17,9 | 45,5 | 130 | 7 | 77,3 | 76,8 | 15,0 | 14,3 | 161,6 | 155,3 | 14,0 | 13,8 | 69,7 | 62,8 | 7,8 | 7,2 |
| 80 | m | 3 | 37 | 33 | 57 | 17 | 56 | 4,9 | 3 | 20,3 | 53,5 | 121 | -20 | 77,1 | 79,6 | 6,7 | 6,4 | 247,5 | 268,0 | 4,2 | 4,6 | 137,9 | 130,1 | 3,6 | 3,6 |
| 81 | m | 2 | 40 | 37 | 59 | 12 | 48 | -5,9 | 4 | 20,5 | 51,0 | 127 | -19 | 81,6 | 83,2 | 10,9 | 10,0 | 183,9 | 186,1 | 11,6 | 11,1 | 104,2 | 100,5 | 7,8 | 7,8 |
| 82 | f | 5 | 43 | 38 | 46 | 3 | 38 | 13,2 | 5 | 19,9 | 50,7 | 126 | -3 | 48,3 | 56,7 | 11,6 | 11,6 | 141,7 | 148,7 | 12,2 | 12,7 | 93,4 | 101,9 | 7,6 | 8,2 |
| 83 | f | 3 | 39 | 36 | 33 | 24 | 26 | -40,9 | 2 | 17,7 | 46,7 | 133 | 6 | 67,6 | 69,2 | 7,1 | 7,1 | 142,2 | 141,2 | 7,1 | 7,7 | 84,2 | 81,3 | 6,6 | 5,8 |
| 84 | m | 7 | 60 | 52 | 73 | 5 | 37 | -5,2 | 4 | 20,8 | 53,0 | 143 | -13 | 98,5 | 98,4 | 8,1 | 8,1 | 236,4 | 239,2 | 6,3 | 6,6 | 147,7 | 168,5 | 5,5 | 6,3 |
| 85 | f | 14 | 55 | 40 | 63 | 9 | 36 | -6,3 | 15 | 17,9 | 47,9 | 131 | -8 | 83,0 | 71,2 | 19,7 | 20,7 | 188,2 | 167,4 | 19,4 | 19,6 | 70,1 | 85,1 | 17,3 | 17,2 |
| 86 | f | 9 | 45 | 36 | 31 | 1 | 21 | 22,7 | 9 | 18,0 | 46,3 | 123 | 14 | 56,7 | 54,4 | 8,4 | 9,5 | 116,2 | 117,9 | 10,4 | 10,4 | 80,7 | 80,8 | 6,6 | 6,8 |
| 87 | m | 0 | 23 | 24 | 57 | 18 | 48 | -9,1 | 1 | 21,1 | 53,4 | 114 | -34 | 78,2 | 88,4 | 6,9 | 6,7 | 213,1 | 228,4 |  |  | 152,0 | 132,0 | 6,4 | 7,0 |
| 88 | f | 11 | 56 | 45 | 66 | 6 | 29 | -22,2 | 8 | 20,7 | 54,0 | 139 | -10 | 67,8 | 72,4 | 8,7 | 8,5 | 144,8 | 134,6 | 25,5 | 24,6 | 120,9 | 124,9 | 6,2 | 5,5 |
| 89 | m | 15 | 49 | 33 | 29 | 9 | 25 | 4,3 | 17 | 20,5 | 54,4 | 122 | 20 | 138,9 | 125,1 | 3,4 | 6,1 | 264,7 | 265,9 | 4,4 | 6,7 | 164,5 | 171,6 | 7,6 | 7,5 |
| 90 | m | 5 | 44 | 38 | 48 | 18 | 45 | -0,4 | 2 | 20,2 | 53,2 | 128 | -4 | 90,8 | 91,9 | 6,4 | 6,9 | 236,6 | 245,6 | 5,2 | 5,5 | 177,7 | 190,7 | 6,2 | 5,6 |
| 91 | m | 21 | 47 | 25 | 46 | 11 | 33 | 18,3 | 22 | 19,5 | 54,0 | 112 | 1 | 118,3 | 116,1 | 4,1 | 5,2 | 271,6 | 232,4 | 4,1 | 5,2 | 215,8 | 208,1 | 0,4 | 1,2 |
| 92 | m | 10 | 40 | 30 | 48 | 18 | 53 | 40,2 | 12 | 19,8 | 50,6 | 115 | -8 | 79,3 | 80,1 | 8,7 | 9,5 | 148,0 | 154,4 | 9,2 | 9,8 | 136,5 | 123,3 | 5,3 | 5,5 |
| 93 | m | 11 | 51 | 40 | 38 | 16 | 40 | 11,5 | 15 | 18,4 | 50,7 | 128 | 13 | 63,6 | 60,1 | 6,6 | 6,4 | 158,0 | 140,7 | 6,8 | 6,8 | 136,4 | 125,7 | 5,9 | 5,3 |
